# Detection of HIV-1 Transmission Clusters from Dried Blood Spots within a Universal Test-and-Treat Trial in East Africa

**DOI:** 10.3390/v14081673

**Published:** 2022-07-29

**Authors:** Emma Pujol-Hodge, Jesus F. Salazar-Gonzalez, Deogratius Ssemwanga, Edwin D. Charlebois, James Ayieko, Heather E. Grant, Teri Liegler, Katherine E. Atkins, Pontiano Kaleebu, Moses R. Kamya, Maya Petersen, Diane V. Havlir, Andrew J. Leigh Brown

**Affiliations:** 1 Ashworth Laboratories, School of Biological Sciences, University of Edinburgh, Edinburgh EH9 3FL, UK; emma.pujol-hodge@ed.ac.uk (E.P.-H.); heather.grant@ed.ac.uk (H.E.G.); 2 Medical Research Council (MRC)/Uganda Virus Research Institute (UVRI) and London School of Hygiene and Tropical Medicine (LSHTM) Uganda Research Unit, Entebbe P.O. Box 49, Uganda; j.sal.gonz.3@gmail.com (J.F.S.-G.); deogratius.ssemwanga@mrcuganda.org (D.S.); pontiano.kaleebu@mrcuganda.org (P.K.); 3 Uganda Virus Research Institute, Entebbe P.O. Box 49, Uganda; 4 Division of Prevention Science, Department of Medicine, University of California, San Francisco, CA 94158, USA; edwin.charlebois@ucsf.edu; 5 Kenya Medical Research Institute, Nairobi P.O. Box 54840-00200, Kenya; jimayieko@gmail.com; 6 Division of HIV, Infectious Diseases and Global Medicine, Department of Medicine, University of California, San Francisco, CA 94110, USA; teri.liegler@ucsf.edu (T.L.); diane.havlir@ucsf.edu (D.V.H.); 7 Usher Institute, University of Edinburgh, Edinburgh EH8 9AG, UK; katherine.atkins@ed.ac.uk; 8 Department of Infectious Disease Epidemiology, Faculty of Epidemiology and Population Health, LSHTM, London WC1E 7HT, UK; 9 Centre for Mathematical Modelling of Infectious Diseases, LSHTM, London WC1E 7HT, UK; 10 School of Medicine, Makerere University, Kampala P.O. Box 7072, Uganda; mkamya@idrc-uganda.org; 11 Division of Biostatistics, School of Public Health, University of California, Berkeley, CA 94720, USA; mayaliv@berkeley.edu

**Keywords:** HIV, phylogenetics, phylodynamics, cluster, transmission network, molecular epidemiology

## Abstract

The Sustainable East Africa Research in Community Health (SEARCH) trial was a universal test-and-treat (UTT) trial in rural Uganda and Kenya, aiming to lower regional HIV-1 incidence. Here, we quantify breakthrough HIV-1 transmissions occurring during the trial from population-based, dried blood spot samples. Between 2013 and 2017, we obtained 549 *gag* and 488 *pol* HIV-1 consensus sequences from 745 participants: 469 participants infected prior to trial commencement and 276 SEARCH-incident infections. Putative transmission clusters, with a 1.5% pairwise genetic distance threshold, were inferred from maximum likelihood phylogenies; clusters arising after the start of SEARCH were identified with Bayesian time-calibrated phylogenies. Our phylodynamic approach identified nine clusters arising after the SEARCH start date: eight pairs and one triplet, representing mostly opposite-gender linked (6/9), within-community transmissions (7/9). Two clusters contained individuals with non-nucleoside reverse transcriptase inhibitor (NNRTI) resistance, both linked to intervention communities. The identification of SEARCH-incident, within-community transmissions reveals the role of unsuppressed individuals in sustaining the epidemic in both arms of a UTT trial setting. The presence of transmitted NNRTI resistance, implying treatment failure to the efavirenz-based antiretroviral therapy (ART) used during SEARCH, highlights the need to improve delivery and adherence to up-to-date ART recommendations, to halt HIV-1 transmission.

## 1. Introduction

**Figure 1:**
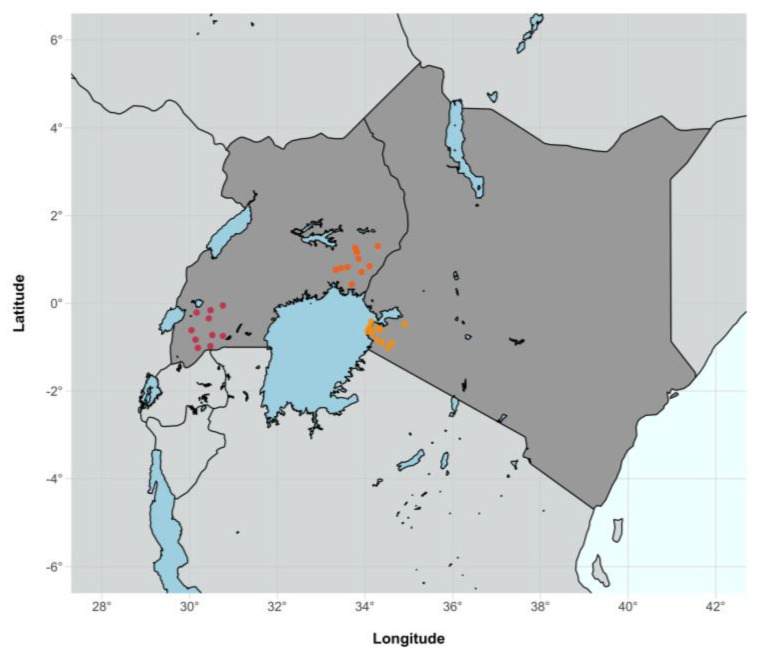
SEARCH community point locations (*n* = 32) across the three geographically separate SEARCH trial regions: Western Uganda (in red, *n* = 10), Eastern Uganda (in orange, *n* = 10), and Western Kenya (in gold, *n* = 12).

The Sustainable East Africa Research in Community Health (SEARCH) trial was a large-scale randomised universal test-and-treat (UTT) trial involving 32 communities in rural Uganda and Kenya (Figure 1), testing the hypothesis that universal HIV treatment and annual testing coupled with a community-based, multi-disease, patient-centred approach would result in a lower number of new HIV infections and better community health in intervention communities [1]. HIV incidence declined in the intervention arm over time, but at three years, incidence did not significantly differ between the intervention and control arms. Population-level HIV testing at baseline and expanded eligibility for universal antiretroviral therapy (ART) in the control arm early in the study contributed to this finding [1]. Results were consistent across other UTT trials, including HPTN 071/PopART in Zambia and South Africa [2], ANRS 12249/TasP in South Africa [3], and BCPP/Ya Tsie in Botswana [4], providing evidence that despite not reaching HIV elimination targets, UTT increases population-level viral suppression [5]. As new HIV infections were reduced but not eliminated in these trials, understanding the dynamics of infection may be able to inform future public health interventions by better targeting of preventative measures.

Phylogenetic studies highlighting HIV-1 transmission dynamics have historically been less common in sub-Saharan Africa due to limited sequence availability [6], but there are increasing efforts to rectify the scarcity. Studies in Uganda, for example, have quantified the history and spread of HIV in the country [7], outlined the role of HIV community-based introductions in their sustainment of rural epidemics [8], and characterised migratory patterns and HIV transmission flows into high-prevalence hotspots from surrounding general population sources [9,10,11,12]. The wealth of information extracted from phylogenetic studies benefits our understanding of HIV-1 infections, evolutionarily and epidemiologically, and with the scaling up of sequencing in sub-Saharan Africa, light will be shed on the transmission networks and dynamics of the region. Phylogenetic knowledge of HIV lineages and their transmission, coupled with epidemiological data, can inform the design of epidemic control measures through our knowledge of transmission events and, thus, their prevention [13].

Here, we report the generation of consensus sequences from partial *gag* and *pol* genes of over 700 participants in the SEARCH trial, using these to detect and characterise HIV-1 transmission clusters among the East Africa SEARCH trial population. SEARCH trial participants were universally screened for HIV at baseline using rapid antibody tests to detect all prevalent HIV-1 infections within the intervention and control communities [1]. However, due to the delayed window of accuracy of rapid antibody tests [14,15], any recent infections (stemming from exposure close to baseline screening) were not detected until a later date and thus classified as incident infections, despite transmission occurring prior to trial initiation. Therefore, we extend our phylogenetic clustering approach to include a phylodynamic analysis of these sequences to distinguish transmissions occurring within the trial from those that predated it. First, we provide an overview of the characteristics of all sequenced SEARCH trial participants, including HIV-1 sequence subtyping and screening for drug-resistant mutations (DRMs). We then identify closely linked sequences using two different approaches: pairwise genetic distance (GD) thresholds to identify any sequences linked at <1.5% GD, and a time-resolved, phylodynamic approach, to identify any linked sequences with an inferred time of transmission arising after the start of the SEARCH trial. By comparing the phylogenetic and phylodynamic approaches, we can identify which putative transmission clusters are relevant in the context of SEARCH, allowing us to understand the nature of incident transmission clusters within a UTT trial setting, to inform better targeted preventative interventions.

## 2. Materials and Methods

### 2.1. Study Design and Population

HIV-1 consensus sequences were obtained from participants in both intervention and control SEARCH trial communities, PCR-amplified from a subset of dried blood spots (DBS), as described by Salazar-Gonzalez et al. [16]. In intervention communities, participants were screened yearly over the three-year trial period (June 2013 to June 2017) to detect incident cases; in control communities, participants were screened twice, at baseline and at the trial end [1]. DBS samples were collected from all SEARCH trial participants, with all incident cases selected for sequencing alongside a convenience sample of baseline cases. Only samples from participants with a measured HIV viral load ≥5000 copies/mL were sent for sequencing.

Specifically, the sequence data consisted of partial HIV-1 *gag* (1.1 kilobases at the 5′-end; *n* = 549) and *pol* (1 kilobase at the 5′-end; *n* = 488) gene sequences from 745 SEARCH trial participants spread across three geographically distinct regions: Western Uganda, Eastern Uganda, and Western Kenya. For 292 SEARCH trial participants, both *gag* and *pol* were sequenced. Epidemiological data were available for all sequenced participants, and characteristics recorded included date of sample collection, participant gender, age, region and community of residence, occupation, and HIV-1 infection category (i.e., prevalent or incident). Incident cases were individuals known to be HIV-1 negative at baseline but whose seroconversion was detected during the SEARCH trial; prevalent cases were those individuals known to be HIV-1 positive at baseline. In total, 730 participants had complete epidemiological data records. Missing values were associated with region, community, and sample collection date (NA = 2), age, and occupation (NA = 15).

### 2.2. Sequence Subtyping

HIV-1 subtyping was carried out using REGAv3 [17] after cross-checking subtype assignments with COMET [18] and SCUEAL [19]. REGAv3 was selected as the subtyping tool of choice as it displayed the most agreement with the other two tools, and it was preferred over the more complex output of SCUEAL subtype assignments due to the purpose of HIV-1 subtyping in this study: to provide an overview of subtype distribution across the SEARCH population, rather than analysing sequence recombination and breakpoints.

### 2.3. Drug Resistance Profiling

The SEARCH *pol* sequences (*n* = 488) were screened for DRMs using the Stanford HIV Drug Resistance Database [20]. Specifically, the sequences were screened for mutations in protease and reverse transcriptase, and drug resistance scores were generated for each of the sequences to the following 20 antiretrovirals (ARVs): abacavir (ABC), zidovudine (AZT), emtricitabine (FTC), lamivudine (3TC), tenofovir (TDF), stavudine (D4T), and didanosine (DDI) (nucleoside reverse transcriptase inhibitors, NRTIs); doravirine (DOR), efavirenz (EFV), etravirine (ETR), nevirapine (NVP), and rilpivirine (RPV) (non-nucleoside reverse transcriptase inhibitors, NNRTIs); atazanavir/ritonavir (ATV/r), darunavir/r (DRV/r), lopinavir/r (LPV/r), fosamprenavir/r (FPV/r), indinavir/r (IDV/r), nelfinavir (NFV), saquinavir/r (SQV/r), and tipranavir/r (TPV/r) (protease inhibitors, PIs).

The prevalence of drug resistance was measured according to World Health Organisation (WHO) standards, where resistance to NNRTI-based ART regimens is defined as resistance to NVP or EFV, resistance to NRTI-based regimens is defined as resistance to any of the NRTIs listed above, resistance to PI-based regimens is defined as resistance to ATV/r, LPV/r or DRV/r, and any resistance is defined as resistance to any of the aforementioned ARVs [21,22]. Only sequences classified as having low-, intermediate-, or high-level resistance were considered for drug resistance prevalence estimates.

### 2.4. Genetic Linkage Analysis

Maximum likelihood phylogenies for the *gag* and *pol* datasets (*n* = 549, *n* = 488, respectively) were constructed using IQ-TREE v1.6.12 [23], and Cluster Picker v1.2.5 (Andrew Leigh Brown Group, University of Edinburgh, Edinburgh, UK) [24] was used to identify clusters (>90% bootstrap support threshold) of closely-related sequences at the <1.5% pairwise GD threshold. To test whether less-supported clusters were being excluded, Cluster Picker was also used to identify any clusters regardless of bootstrap support at the <1.5% GD threshold; however, the same clusters were detected, indicating high support for the identified clusters.

Cluster Picker was favoured over other cluster detection tools, such as HIV-TRACE [25], as a result of findings from Rose and colleagues [26]. Briefly, the different clustering algorithms used by Cluster Picker and HIV-TRACE (maximum pairwise GD and single-linkage clustering, respectively) result in Cluster Picker better detecting distinct, two-individual transmission events separated by long time periods allowing for viral population divergence, and HIV-TRACE better detecting larger and fewer transmission clusters, which may be advantageous for detection of larger outbreaks or epidemics with high coverage [26]. Consequently, due to the small proportion of sequenced SEARCH trial participants, Cluster Picker remained the better option to identify transmission clusters in our dataset.

As both *gag* and *pol* sequences were available for 292 trial participants, there was some overlap between clusters identified for each dataset, resulting in some clusters being counted twice. Moreover, some participants for whom both genes were sequenced were only in *gag* or *pol* clusters, resulting in them being excluded from analysis due to not being a conclusive cluster.

In R v4.0.2 (https://cloud.r-project.org/index.html, accessed on 10 May 2022) [27], a logistic, stepwise regression model was set up to determine whether certain characteristics made sequenced SEARCH participants more likely of being in a <1.5% GD cluster, and adjusted odds ratios were calculated for statistically significant variables. The best-fitting model (AIC: 227.54, Table S1) included region, occupation, HIV-1 infection category, and SEARCH trial arm.

### 2.5. Phylodynamic Analysis

Only inferred transmission clusters arising after the start of the SEARCH trial (June 2013) were of public health interest in this study, which aimed to investigate where new infections during the SEARCH trial stemmed from. To estimate the time to the most recent common ancestor (tMRCA) and ages of any internal nodes in SEARCH-incident clusters (used as proxies for cluster ages and within-cluster transmission events, respectively), we conducted Bayesian molecular clock phylogenetic inference using BEAST v1.10.4 (http://www.beast2.org/, accessed on 10 May 2022) [28]. To measure ancestral node estimates more accurately, historical HIV-1 sequences were included in determining a calibration rate for the molecular clock. Sequences were included from two historical timepoints: 1998 and 1999 published HIV-1 Ugandan sequences [29] and 1986 Ugandan sequences from our group [30].

After removing any temporal sequence outliers [31] using TempEst v1.5.3 (http://tree.bio.ed.ac.uk/software/tempest/, accessed on 10 May 2022) [32], four different BEAST runs with SEARCH and historical sequences were set up, accounting for uncertainty where incomplete sampling dates were included: *gag* A1 (*n* = 339), *gag* D (*n* = 116), *pol* A1 (*n* = 297), and *pol* D (*n* = 102). All runs were performed under an uncorrelated relaxed log-normal molecular clock [33] for 1 billion generations, sampling every 10,000th generation. The SRD06 nucleotide substitution model was used for all runs, which consists of the HKY substitution model [34] with four categories of rate heterogeneity (γ + 4) [35] and two codon position partitions that are parameterised separately: positions (1 + 2) and position 3. The coalescent tree prior differed according to subtype: for subtype A1 runs, a Bayesian SkyGrid prior [36] with 20 parameters and a time at the last transition point of 80 was used; for subtype D runs, a GMRF Bayesian Skyride prior [37] was used, and the clock rate was allocated a narrow, truncated prior of mean 0.003 substitutions/site/year (a normal distribution with a standard deviation of 0.01, in [0.001, 0.01]).

The aforementioned BEAST priors were selected after assessing convergence and mixing of a variety of runs (see Table S2 and Figures S1–S3) in Tracer v1.7.1 (http://tree.bio.ed.ac.uk/software/tracer/, accessed on 10 May 2022) [31,38]. For the final four runs selected, the posterior distribution trace plots were indicative of efficient Markov chain Monte Carlo chains with good mixing [39], and effective sample size (ESS) values were above 200 for all parameters in *gag* D, *pol* A1, and *pol* D runs (and all ESS values above 150 for *gag* A1). Additionally, runs were selected that minimised the width of the 95% highest posterior density intervals (95% HPD) to minimise the error surrounding internal node age estimates.

TreeAnnotator v1.10.4 (https://beast.community/treeannotator, accessed on 10 May 2022), distributed as part of the core BEAST package, was used to generate maximum clade credibility (MCC) trees with median node heights for each of the BEAST runs, discarding 10% of the states as burn-in. Using the MCC trees, the median age and 95% HPD for each node in a SEARCH-incident cluster were extracted, with each representing a maximum estimate of the time for the inferred transmission event.

In R v4.0.2 [27], a logistic, stepwise regression model was set up to determine whether certain characteristics made sequenced SEARCH participants more likely to be in SEARCH-incident clusters, and adjusted odds ratios were calculated for statistically significant variables. The best-fitting model (AIC: 142.79, Table S3) included occupation and HIV-1 infection.

## 3. Results

### 3.1. Characteristics of Sequenced SEARCH Trial Participants

The epidemiological characteristics of sequenced, HIV-1 positive SEARCH trial participants (*n* = 745) are summarised in Table 1, Tables S4 and S5, according to the SEARCH geographical region, trial arm, and HIV-1 infection category (prevalent or incident), respectively. The age and occupation of sequenced participants were categorised using the same classification as the seminal SEARCH trial results [1]. Of the sequenced participants, 410 (55%) were female, and 469 (63%) were classified as prevalent infections (known to be HIV-infected at SEARCH trial baseline, either through rapid antibody testing or documented HIV-positive from Ministry Record). Over half of sequenced participants (376, 50.5%) reported their occupation as farmers, a “low-risk informal sector” occupation. After farmers, healthcare workers (69, 9.3%) and household workers (69, 9.3%) were the most common occupations. The median age of sequenced participants was 33, ranging from 17 to 86 years.

### 3.2. Sequencing Density

At baseline, 13,529 SEARCH trial participants were reported as living with HIV: 2873 (21.2%) in Western Uganda, 1590 (11.7%) in Eastern Uganda, and 9066 (67%) in Western Kenya [1]. However, owing to differences in success rate from DBS sequencing, this distribution is not well represented by the prevalent (i.e., HIV-1 positive at baseline) sequenced subset of participants: 263 sequences (56.1%) from Western Uganda, 5 (1.1%) from Eastern Uganda, and 199 (42.4%) from Western Kenya. With regard to incident cases, 850 were reported over the course of the trial: 317 (37.3%) in Western Uganda, 132 (15.5%) in Eastern Uganda, and 401 (47.2%) in Western Kenya. The geographical distribution of the 276 sequenced incident cases was 114 (41.3%), 56 (20.3%), and 106 (38.4%), respectively, and was approximately in line with the overall incident cases recorded during the trial, albeit with an underrepresentation of Kenyan incident cases.

**Figure 2:**
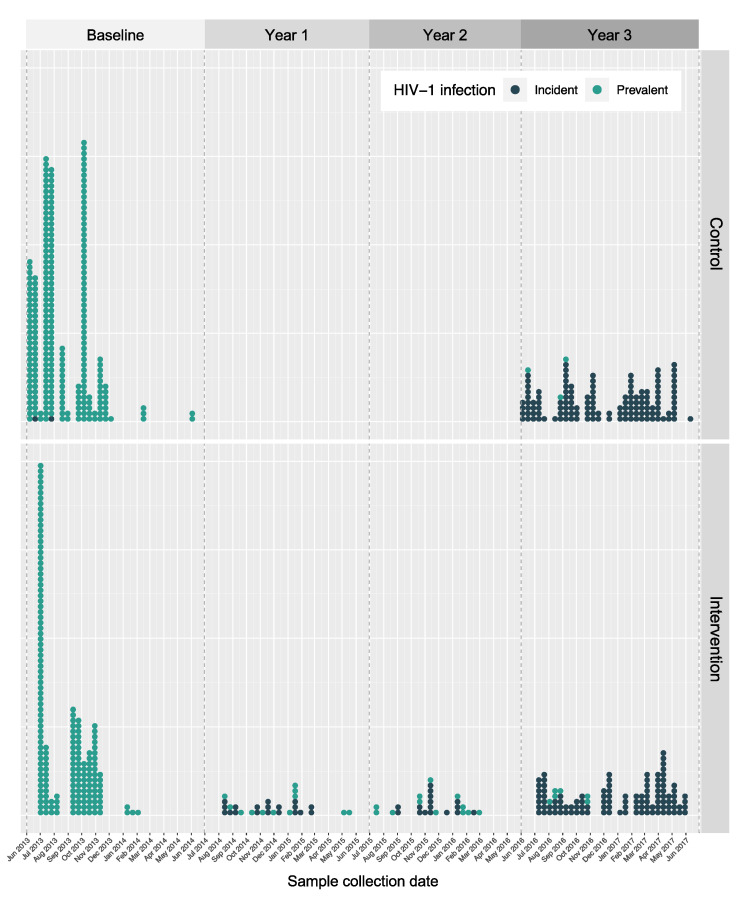
Sequencing intensity dot plot of the control (top) and intervention (bottom) arms of the SEARCH trial, with distinction between incident (dark) and prevalent (light) infections. Screening frequency differed between the intervention and control arms: yearly screening in intervention communities; baseline and year 3 screening only in control communities. Two incident infections were detected during rounds of baseline testing; most prevalent infections were detected at baseline, with the remaining picked up in later rounds.

The prevalent and incident sequencing densities (the percentage of HIV-1 positive SEARCH trial participants successfully amplified) are summarised in Table 2, according to geographical region and the SEARCH trial arm. Overall, the sequencing density of incident HIV-1 infections is much higher (above 30% in all cases) than the sequencing density of prevalent HIV-1 infections, highlighting the priority of the SEARCH trial with regard to sequencing incident infections recorded during the trial. The timing of sample collection for sequenced participants, with distinction between the trial arm and HIV-1 infection category, is summarised in Figure 2, showcasing the difference in screening intensity between intervention and control communities.

### 3.3. Subtype Distribution

SEARCH *gag* (*n* = 549) and *pol* (*n* = 488) sequences were classified as HIV-1 subtypes (A1, A2, C, D, or G) or inter-subtype recombinants (recombinants) using REGAv3 [17], and the regional distribution of subtypes for each gene sequence is summarised in Table 3. Overall, the most prevalent HIV-1 subtype was A1 in both *gag* and *pol,* with 323 (58.8%) and 278 (57%) sequences, respectively. This was followed by subtype D, also in both genes, and by recombinants. The distribution of subtypes was broadly similar between *gag* and *pol,* with a slightly higher number of recombinants and a lower number of subtype A1 sequences in *pol*. When stratified by geographical region (Table 3), the distribution of subtypes varied: the frequency of subtype D was higher in Western and Eastern Uganda compared to Western Kenya (~20% vs. ~10%, respectively), subtype A1 was higher in eastern regions (Eastern Uganda and Western Kenya, ~60–70% vs. 50% in Western Uganda), subtype A2 was only identified in Western Kenya, and Eastern Uganda presented lower subtype diversity. Subtype distribution differed the most between Western Uganda and Western Kenya, with χ^2^ test of association *p*-values of 1.1 × 10^−5^ and 4.6 × 10^−4^ for *gag* and *pol* subtype distributions, respectively, while differences between both Ugandan regions and Eastern Uganda and Western Kenya were more nuanced and depended on the gene under consideration (Table 3).

### 3.4. Drug Resistance Profiles

Participants sequenced in the *pol* region (*n* = 488) were screened for drug resistance. Overall, 69 (14.1%) sequences were resistant (according to WHO criteria, see *Methods*). When stratified by drug class, resistance to NNRTI-based regimens was the most prevalent, with 66 (13.5%) sequences exhibiting NNRTI resistance, followed by 26 (5.3%) sequences with resistance to NRTI-based regimens. No PI resistance was identified in the sample. Resistance stratified by the SEARCH trial arm and HIV-1 infection category is summarised in Table 4. Based on χ^2^ tests of association, no significant difference was found in the distribution of drug-resistant and drug-sensitive *pol* sequences according to geographical region (*p*-value = 0.51), SEARCH trial arm (*p*-value = 0.5), or prevalent/incident HIV-1 infections (*p*-value = 0.63).

Within the resistant sequences identified, the most common NNRTI-associated mutation was K013N (*n* = 37), with an overall prevalence of 7.6% in *pol* SEARCH sequences (95% CI = 5.4–10%), followed by Y181C (*n* = 8; 1.6%, 95% CI = 0.7–3.2%), and G190A (*n* = 7; 1.4%, 95% CI = 0.6–2.9%). The most common NRTI-associated mutation was M184V (*n* = 16; 3.3%, 95% CI = 1.9–5.3%), followed by D67N and M41L (*n* = 4; 0.8%, 95% CI = 0.2–2.1%, both). A full list of the 69 resistant sequences alongside their resistance levels and associated mutations is available in Table S6.

### 3.5. Identification of SEARCH Trial Clusters

#### 3.5.1. Genetically Linked Clusters

**Figure 3:**
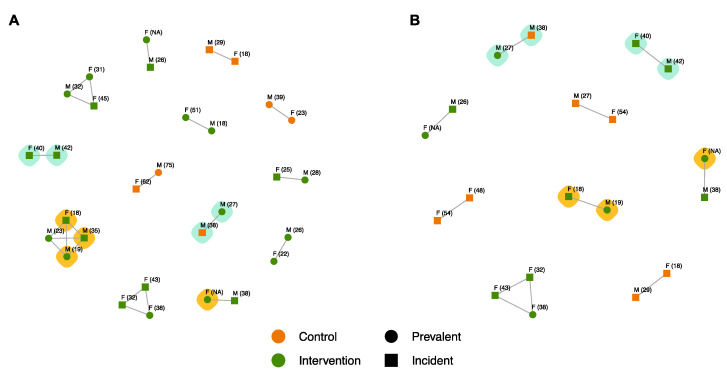
(**A**) SEARCH sequenced participants in <1.5% pairwise genetic distance (GD) clusters (*n* = 30 sequences) and (**B**) participants in SEARCH-incident, phylodynamic clusters (*n* = 19 sequences). Nodes representing participants from control communities are in orange, intervention communities are in green; circles represent prevalent infections, squares incident infections; nodes are labelled with the gender (M = male, F = female) and age (years, in brackets) for each participant; turquoise-highlighted clusters are inferred inter-community transmissions, gold-highlighted sequences are NNRTI resistant.

Maximum likelihood phylogenies were generated using IQ-TREE v1.6.12 [23] and analysed using Cluster Picker v1.2.5 [24] to identify clusters of closely related sequences with a maximum pairwise GD of 1.5%. We identified 13 unique <1.5% GD clusters, ranging in size from two to four sequenced participants, summarised in Figure 3A and Table 5. Of the 13 clusters, 11 (84.6%) contained at least one male and one female participant, 11 (84.6%) were within-community clusters, 10 (76.9%) contained at least one incident participant, and 9 (69.2%) were intervention community clusters. Most clusters (8 clusters, 61.5%) were between participants from within the same age category. The odds of being in a cluster were lower for sequenced participants with “low-risk informal sector” occupations, with an adjusted odds ratio (aOR) of 0.34 and a 95% CI of 0.14–0.83%. The odds were higher for sequenced participants from intervention communities (aOR = 3.34, 95% CI = 1.42–8.79%). There were no other characteristics recorded that differed significantly between clustered and non-clustered sequenced participants.

#### 3.5.2. SEARCH-Incident Transmission Events

In the above-described phylogeny-based analysis, three of the <1.5% GD clusters identified were recorded within the trial as prevalent pairs (Figure 3A), i.e., the inferred transmission event that generated the cluster occurred before the implementation of SEARCH interventions. To characterise clusters arising after the start of the SEARCH trial, we adopted an approach based on a time-resolved, phylodynamic methodology to exclude genetically linked clusters with an inferred transmission time prior to the start of SEARCH. We used BEAST v1.10.4 [28], which incorporates a locally autocorrelated clock in a Bayesian phylodynamic framework, to estimate the tMRCA for all linked sequences. From the time-resolved trees generated (Figures S2 and S3), we identified nine incident SEARCH clusters, eight pairs and one triplet, summarised in Figure 3B and Table 5.

The characteristics of the clusters changed somewhat when restricted to the period of the trial. Overall, six (66.7%) were differing sex clusters, seven (77.8%) were within-community clusters, all contained at least one incident participant, and five (55.6%) were intervention community clusters. The odds of being in a cluster were lower for sequenced participants with “low-risk informal sector” occupations (aOR = 0.16, 95% CI = 0.05–0.46%), and higher for sequenced participants with incident infections (aOR = 7.78, 95% CI = 2.48–34.2%). No other recorded characteristics were significantly different among sequenced participants in SEARCH-incident clusters and sequenced HIV-positive participants not in clusters.

### 3.6. Drug Resistance Characterisation of SEARCH Trial Clusters

For the seven unique phylodynamic clusters with *pol*-sequenced participants, sequences were screened for drug resistance (Figure 3B). One pair of subtype D sequences was identified with both sequences highly resistant to NNRTIs (K103N mutation), which were closely linked to a third sequence with the same high-resistance mutation. This triplet shared a recent common ancestor, with a median age prior to the start of the SEARCH trial but with a 95% HPD overlapping the SEARCH trial timeline, and lay within the genetically linked, four-person cluster depicted in Figure 3A. These participants were all from the same intervention community in Western Uganda: those in the drug-resistant SEARCH-incident cluster were an 18-year-old incident female and a 19-year-old prevalent male, while the closely linked third participant was a 35-year-old incident male. Separately, a subtype A1 sequence from a prevalent female of unknown age was found to also be highly resistant to NNRTIs with a K103N mutation, although their cluster pair (a 38-year-old incident male from the same Kenyan intervention community) was not, suggesting resistance was not transmitted in this case. Overall, the prevalence of resistance amongst participants in SEARCH-incident clusters was 18.8%, while the prevalence of resistance amongst non-clustering participants was 14%; however, this difference was not statistically significant (χ^2^ test of association *p*-value = 0.86).

## 4. Discussion

The aim of this study was to detect and characterise HIV-1 transmission clusters among the East Africa SEARCH trial population, with particular interest in transmission events which occurred after the start of the trial. The study included 745 HIV-1 positive SEARCH trial participants, from which partial HIV-1 *gag* and/or *pol* consensus sequences were available. Among sequenced participants, the most prevalent subtype was A1 in all geographical regions, followed by subtype D. Over a quarter (25.2%) of HIV-1 sequences had at least one DRM, and 14.1% of sequences had resistance to at least one class of ARVs. Using phylogenetic methods, 13 clusters were identified with <1.5% pairwise GD. Applying phylodynamic approaches to the analysis enabled the identification of nine clusters with an inferred tMRCA later than the start of the SEARCH trial, deemed SEARCH-incident clusters. The identification of SEARCH-incident clusters is an indicator of ongoing HIV-1 transmission within a UTT trial setting, where substantial efforts were made to reduce transmission.

### 4.1. Regional Distribution of HIV-1 Subtypes

The regional distribution of HIV-1 subtypes highlights the differences in subtype diversity between Uganda and Kenya, such as the higher proportion of subtype D sequences in Uganda (both East and West) compared to Western Kenya, where the proportion of subtypes A1 and A2 is higher (Table 3). Our results are broadly consistent with published literature on HIV-1 subtype distribution in East Africa, with regard to the higher prevalence of subtypes A and D [40,41,42] and the prevalence of HIV-1 recombinant forms in East Africa [43]. Eastern Uganda presented lower subtype diversity: no subtype C was identified, in contrast with the findings of Poon and colleagues [42], who reported a low frequency of subtype C in the region. Nevertheless, the lower diversity of Eastern Ugandan sequences is likely due to the smaller regional sample size (61 sequenced SEARCH trial participants, compared to 377 and 305 for Western Uganda and Western Kenya, respectively). Overall, our results indicate that despite the small size, the sequenced sample of SEARCH trial participants is representative of the HIV-1 subtype distribution in East Africa.

### 4.2. Drug Resistance in SEARCH

The type of DRMs identified in our dataset are amongst the most commonly identified in East Africa [44]: M184V is the most prevalent NRTI mutation in East Africa and SEARCH; K103N, Y181C, and G190A are the most prevalent NNRTIs in East Africa, with K103N the most prevalent in the SEARCH sample, followed by Y181C and G190A (Table S6). Discrepancies in the type of mutation and its prevalence in the SEARCH trial compared to ref. [44] were also found. For PI mutations, East Africa prevalence estimates suggest I85V and N88D are the most common; however, neither mutation was identified in SEARCH, likely due to the lack of power owing to the lower overall prevalence of PI mutations and the sequenced sample size.

The identified prevalence of drug resistance across sequenced SEARCH trial participants was 14.1%, with 25.2% of sequences harbouring at least one DRM. Drug resistance was highest to NNRTIs, followed by NRTIs and PIs, consistent with published literature [21,22,44]. For Uganda specifically, where data on national resistance prevalence is available for 2016 [21], the levels of drug resistance amongst Ugandan SEARCH trial participants were lower than the national estimates; however, the differences were not statistically significant (χ^2^ test of association *p*-value = 0.38): 17.4% vs. 13.1% for any drug resistance; 15.4% vs. 12.4% for NNRTI resistance; 5.1% vs. 4.9% for NRTI resistance; 1% vs. none for PI resistance.

The acquisition and transmission of DRMs reflects a population where prescribed ART regimens, either through national guidelines or via the SEARCH trial, were not suppressing viral replication, resulting in downstream effects on HIV-1 transmission. The detection of DRMs in treatment naïve incident cases further highlights this issue. The high prevalence of NNRTI resistance, particularly of sequences harbouring resistance mutation K103N, highlights issues with the first-line EFV-based regimens administered during the SEARCH trial, for which K103N reduces susceptibility 20-fold [20].

No differences were found in the distribution of drug-resistant and drug-sensitive sequences between trial arms; nevertheless, a lower level of transmitted drug resistance could have been expected in intervention communities due to earlier universal ART provision and increased monitoring.

### 4.3. The Effect of the SEARCH Sampling Frequency on Genetic Linkage Clustering

Using the <1.5% GD threshold for clustering, we estimated that the adjusted odds of being in a cluster were 3.34 times higher for participants from SEARCH intervention communities compared to control communities. This is likely an artefact of differential sampling between trial arms: with intervention community sampling performed yearly throughout the course of the trial, incident cases are detected at an earlier timepoint after infection onset, compared to incident infections in control communities which are only detected at the end of the trial, as sampling was performed at baseline and in the final year of SEARCH only. This differential sampling frequency, coupled with HIV-1 mutation rates, results in a higher chance of <1.5% GD clusters being detected in intervention communities than in control communities due to shorter sequence divergence times between infection and detection of incident cases. Indeed, simulation analyses demonstrated that clustering methods are systematically biased to detect variation in sampling rates rather than transmission rate variation [45].

The difficulty in addressing the sampling difference with a clustering approach led to the adoption of the more demanding phylodynamic approach, through which we would be able to distinguish estimated infection times as being within or outside the period of the trial.

Increased odds of clustering for intervention community participants were not observed when using a phylodynamic approach for cluster detection. This confirms that the finding is a sampling artefact. As our priority was to detect transmission clusters arising during the SEARCH trial, by assigning ages to the ancestral nodes we were able to exclude clusters with inferred ages prior to the start of the trial, removing <1.5% GD clusters that did not meet this criterion.

### 4.4. Inferred SEARCH Trial Transmission Events

Overall, nine unique clusters were identified with a median tMRCA after the start of the SEARCH trial, representing transmission events occurring during the trial (Table 5). Moreover, when considering whether the tMRCA 95% HPD interval for a cluster with incident cases overlapped with the start of the SEARCH trial, an additional four clusters (three control, one intervention) were identified, and an intervention community cluster was expanded from two to four members.

Of the nine unique clusters with SEARCH-incident start date medians, three were same-gender clusters: two all-female and one all-male cluster (Figure 3B, Table 5). The all-female clusters likely represent an unsampled, “missing” male, especially as there was higher female enrolment in the SEARCH trial, while the male–male pair could indicate transmission among men who have sex with men or an unsampled common female sexual partner.

Both inferred inter-community transmission events identified were between Kenyan communities. This aligns with the findings of Camlin and colleagues, who quantified HIV-associated mobility within the same SEARCH geographical regions and found the highest mobility was in communities from Western Kenya [46]. We found 22.2% of transmission events to represent inferred inter-community transmissions, contrasting with the reported 40% prevalence of inter-community phylogenetic clusters in rural Rakai District, in southwestern Uganda [8]. This apparent variability in inter-community transmission estimates among different study populations highlights differences in study design, where SEARCH communities were designed to be geographically separated, and suggests a highly context-specific feature to HIV-associated mobility and transmission measurement. Notwithstanding, the identified phylodynamic clusters reflect the rural dynamics of HIV-1 transmission in East Africa: mainly heterosexual, within-community transmissions.

The adjusted odds of being in a SEARCH-incident phylodynamic cluster were 7.78 higher for participants with incident HIV-1 infections. This is not unexpected, as participants identified with incident infections in the context of the SEARCH trial are at most three years into an infection. Given that most sources of infection would lie within the SEARCH communities, among which recruitment of prevalent cases was very high, the probability of clustering will be close to the probability at which prevalent cases were sequenced. However, the probability of a prevalent case being included is reduced by the much lower probability at which prevalent cases receiving ART transmit HIV.

Two highly NNRTI-resistant clusters, both with K103N mutations, were identified within SEARCH-incident clusters, one of which possibly highlights evidence of EFV-based drug resistance transmission within the SEARCH trial. While K103N is one of the most frequently transmitted DRMs in East Africa [44] and severely reduces the efficacy of the NNRTIs in widespread use in sub-Saharan Africa, identification, and possible transmission, of such mutation within the highly monitored context of the SEARCH trial emphasises the importance of improved ART delivery, regimens, comprehensive treatment access and adherence, and intensified viral load monitoring to reduce the spread of resistance in rural East African populations [47]. With EFV-based regimens being phased out, adherence to the currently recommended ART regimen of DTG, 3TC, and TDF [48] will continue to play an important role in reducing HIV transmission and preserving health, particularly in light of the higher barrier to DTG drug resistance development [49].

### 4.5. Limitations

Our study has several limitations. Firstly, the low fraction of all HIV-1 positive SEARCH trial participants that were sequenced, along with the difference in sampling density for prevalent and incident infections, resulted in missing data with regard to transmission cluster analysis, both phylogenetic and phylodynamic. This consequently leads to an incomplete picture of the SEARCH trial HIV-1 epidemic, complicating the interpretation of results.

At the time the SEARCH trial was planned, phylogenetic analysis was not being used to track onward transmission. Nevertheless, HIV-1 consensus sequences were obtained from DBS stored during the trial and used here to provide important insights into transmission dynamics among trial participants. There were challenges involving the successful amplification of DBS samples, consequently resulting in a lower number of sequences obtained. In future work, suitable trial sample storage would allow more detailed investigation, for example, using deep sequencing reads [12,50].

## 5. Conclusions

Our study provides insights into the characteristics and dynamics of HIV-1 transmissions within the East Africa SEARCH trial population. Our results corroborate findings on the distribution of HIV-1 subtypes and drug resistance in Uganda and Kenya. Phylodynamic cluster characterisation indicated how most HIV-1 transmissions during the SEARCH trial were within the same community; however, almost one-quarter of transmissions were inferred to originate outside the community, highlighting the role of targeting mobile populations to halt inter-community spread. Monitoring cluster formation and tailoring prevention and risk reduction interventions to persons with common characteristics to those of clustering incident infections will allow more rapid interruption of transmission. Altogether, these efforts will contribute to reducing the incidence of HIV-1 infections in the ongoing East African epidemic as the field moves forward with advances in ART treatments and prevention.

## Figures and Tables

**Table 1:** Characteristics of sequenced SEARCH trial participants by geographical region (*n* = 745).

	Western Uganda	Eastern Uganda	Western Kenya	NA	Total
	*n* (%)	*n* (%)	*n* (%)	*n* (%)	*n* (%)
***All***	377 (50.6)	61 (8.2)	305 (40.9)	2 (0.3)	745 (100)
***Gender***					
(χ^2^ test *p*-value = 5 × 10^−5^)					
Female	180 (47.7)	31 (50.8)	197 (64.6)	2 (100)	410 (55)
Male	197 (52.3)	30 (49.2)	108 (35.4)	0	335 (45)
***Age category***					
15–20 years	17 (4.5)	4 (6.6)	18 (5.9)	0	39 (5.2)
21–49 years	311 (82.5)	47 (77)	236 (77.4)	0	594 (79.7)
≥50 years	45 (11.9)	10 (16.4)	42 (13.8)	0	97 (13)
NA	4 (1.1)	0	9 (3)	2 (100)	15 (2)
***Occupation*** *					
(χ^2^ test *p*-value = 7 × 10^−4^)					
Formal sector	50 (13.3)	6 (9.8)	66 (21.6)	0	122 (16.4)
High-risk informal sector	22 (5.8)	4 (6.6)	24 (7.9)	0	50 (6.7)
Low-risk informal sector	282 (74.8)	51 (83.6)	179 (58.7)	0	512 (68.7)
Other	12 (3.2)	0	20 (6.6)	0	32 (4.3)
No job or disabled	7 (1.9)	0	7 (2.3)	0	14 (1.9)
NA	4 (1.1)	0	9 (3)	2 (100)	15 (2)
***HIV-1 infection*** ^†^					
(χ^2^ test *p*-value < 2 × 10^−16^)					
Prevalent	263 (69.8)	5 (8.2)	199 (65.2)	2 (100)	469 (63)
Incident	114 (30.2)	56 (91.8)	106 (34.8)	0	276 (37)
***Trial arm***					
(χ^2^ test *p*-value = 6 × 10^−9^)					
Intervention	142 (37.7)	22 (36.1)	184 (60.3)	0	348 (46.7)
Control	235 (62.3)	39 (63.9)	121 (39.7)	0	395 (53)
NA	0	0	0	2 (100)	2 (0.3)

* A *formal sector* occupation was defined as a teacher, student, government worker, military worker, health worker, or factory worker. A *high-risk informal sector* occupation was defined as a fishmonger, fisher, bar owner, bar worker, transportation worker, or factory worker. A *low-risk informal sector* occupation was defined as a farmer, shopkeeper, market vendor, hotel worker, homemaker, household worker, construction worker, or miner. ^†^ *HIV-1 infection* was defined as *prevalent* if HIV-1 positive at SEARCH trial baseline and *incident* if HIV-1 negative at baseline but if seroconversion was detected during the trial.

**Table 2:** Prevalent and incident sequencing densities by geographical region and by trial arm.

	Total HIV+	Sequenced HIV+	Sequencing Density
	*n*	*n*	%
***Prevalent* sequencing density by *geographical region***			
Western Uganda	2873	263	9.15
Eastern Uganda	1590	5	0.31
Western Kenya	9066	199	2.19
**Total**	13,529	467	3.45
***Incident* sequencing density by *geographical region***			
Western Uganda	317	114	35.96
Eastern Uganda	132	56	42.42
Western Kenya	401	106	26.43
**Total**	850	276	32.47
***Prevalent* sequencing density by *trial arm***			
Intervention	7212	204	2.83
Control	6317	263	4.16
**Total**	13,529	467	3.45
***Incident* sequencing density by *trial arm***			
Intervention	435	144	33.10
Control	415	132	31.81
**Total**	850	276	32.47

**Table 3:** *gag* (*n* = 548, NA = 1) and *pol* (*n* = 486, NA = 2) HIV-1 subtype distribution by region.

	Western Uganda		Eastern Uganda		Western Kenya		Total	
	*n* (%)		*n* (%)		*n* (%)		*n* (%)	
	*gag*	*pol*	*gag*	*pol*	*gag*	*pol*	*gag*	*pol*
A1	141 (49.1)	122 (48.8)	32 (68.1)	35 (62.5)	149 (69.6)	119 (66.1)	322 (58.8)	276 (56.8)
A2	0	0	0	0	4 (1.9)	3 (1.7)	4 (0.7)	3 (0.6)
D	71 (24.7)	61 (24.4)	10 (21.3)	14 (25)	25 (11.7)	21 (11.7)	106 (19.3)	96 (19.7)
C	29 (10.1)	20 (8)	0	0	12 (5.6)	8 (4.4)	41 (7.5)	28 (5.8)
G	1 (0.4)	1 (0.4)	0	0	2 (0.9)	0	3 (0.6)	1 (0.2)
Recombinants	45 (15.7)	46 (18.4)	5 (10.6)	7 (12.5)	22 (10.3)	29 (16.1)	72 (13.1)	82 (16.9)
**Total**	287 (100)	250 (100)	47 (100)	56 (100)	214 (100)	180 (100)	548 (100)	486 (100)

To determine subtype diversity, χ^2^ tests of association were conducted comparing the distribution of A1, D, and other (A2, C, G, and recombinants) sequences between regions: across all three regions, *gag p*-value = 2.5 × 10^−5^, *pol p*-value = 6.2 × 10^−4^; between Western Uganda and Eastern Uganda, *gag p*-value = 0.03, *pol* *p*-value = 0.06; between Western Uganda and Western Kenya, *gag p*-value = 1.1 × 10^−5^, *pol* *p*-value = 4.6 × 10^−4.^; between Eastern Uganda and Western Kenya, *gag* *p*-value = 0.13, *pol p*-value = 0.03.

**Table 4:** Drug resistant sequences, by trial arm (*n* = 486, NA = 2 *) and HIV-1 infection ^†^ (*n* = 488).

	Sensitive	NRTI Resistance Only	NNRTI Resistance Only	NRTI + NNRTI Resistance	Total
	*n* (%)	*n* (%)	*n* (%)	*n* (%)	*n* (%)
**Drug resistant sequences by *trial arm***					
Intervention	203 (87.1)	2 (0.9)	27 (11.6)	1 (0.4)	233 (47.9)
Control	214 (84.6)	1 (0.4)	34 (13.4)	4 (1.6)	253 (52.1)
**Total**	417 (85.8)	3 (0.6)	61 (12.6)	5 (1)	486 (100)
**Drug resistant sequences by *HIV-1 infection*** ^†^					
Prevalent	208 (84.9)	2 (0.8)	31 (12.7)	4 (1.6)	245 (50.2)
Incident	211 (86.8)	1 (0.4)	30 (12.3)	1 (0.4)	243 (49.8)
**Total**	419 (85.9)	3 (0.6)	61 (12.5)	5 (1)	488 (100)

*NRTI* = nucleoside reverse transcriptase inhibitor; *NNRTI* = non-nucleoside reverse transcriptase inhibitor. * Two sensitive *pol* sequences were unallocated to control or intervention communities. ^†^ *HIV-1 infection* was defined as *prevalent* if HIV-1 positive at SEARCH trial baseline and *incident* if HIV-1 negative at baseline but if seroconversion was detected during the trial. Incident sequences are ART-naïve at sampling; prevalent sequences may be ART-experienced at sampling.

**Table 5:** Characteristics of SEARCH <1.5% pairwise genetic distance (GD) clusters (*n* = 13) and of phylodynamic clusters arising after the start of the SEARCH trial (*n* = 9).

	<1.5% GD Clusters	SEARCH-Incident Clusters
	*n* (%)	*n* (%)
***All***	13 (100)	9 (100)
***Gender***		
Differing gender cluster	11 (84.6)	6 (66.7)
Same gender (female) cluster	1 (7.7)	2 (22.2)
Same gender (male) cluster	1 (7.7)	1 (11.1)
***HIV-1 infection category***		
Prevalent cluster	3 (23.1)	0
Incident cluster	2 (15.4)	4 (44.4)
Mixed cluster	8 (61.5)	5 (55.6)
***Region***		
Intra-region cluster	13 (100)	9 (100)
Inter-region cluster	0	0
***Community***		
Intra-community cluster	11 (84.6)	7 (77.8)
Inter-community cluster	2 (15.4)	2 (22.2)
***Age***		
Intra-age cluster	8 (61.5)	4 (44.4)
Inter-age cluster	3 (23.1)	3 (33.3)
NA	2 (15.4)	2 (22.2)
***Occupation***		
Intra-occupation cluster	4 (30.8)	3 (33.3)
Inter-occupation cluster	7 (53.8)	4 (44.4)
NA	2 (15.4)	2 (22.2)
***Trial arm***		
Intervention cluster	9 (69.2)	5 (55.6)
Control cluster	3 (23.1)	3 (33.3)
Mixed cluster	1 (7.7)	1 (11.1)
***Subtype***		
A1 cluster	10 (76.9)	7 (77.8)
D cluster	2 (15.4)	2 (22.2)
Recombinant cluster	1 (7.7)	0

## Data Availability

Sequence data analysed in this study have been submitted to GenBank under accession numbers ON501128-ON502164.

## Supplementary Materials

The following supporting information can be downloaded at: https://www.mdpi.com/article/10.3390/v14081673/s1, Table S1: Estimated regression parameters, standard errors, *z*-values, and *p*-values for the logistic regression model used to determine whether certain characteristics made sequenced SEARCH participants more likely of being in a <1.5% genetic distance (GD) cluster; Table S2: Settings for tested BEAST runs [31,33,34,35,36,37,51,52,53]; Figure S1: Median node ages and associated 95% highest posterior density (95% HPD) intervals for SEARCH-incident nodes; Figure S2: Maximum clade credibility trees with median node ages for *gag* A1 (left) and *gag* D (right) SEARCH trial sequences; Figure S3: Maximum clade credibility trees with median node ages for *pol* A1 (left) and *pol* D (right) SEARCH trial sequences; Table S3: Estimated regression parameters, standard errors, *z*-values, and *p*-values for the logistic regression model used to determine whether certain characteristics made sequenced SEARCH participants more likely of being in SEARCH-incident clusters; Table S4: Characteristics of sequenced SEARCH trial participants, by trial arm (*n* = 745); Table S5: Characteristics of sequenced SEARCH trial participants, according to HIV-1 infection category (*n* = 745); Table S6: SEARCH trial sequences with low- to high-level NRTI/NNRTI resistance, according to WHO standards for resistance classification, alongside NRTI/NNRTI- associated mutations.
