# Supplementary material for: Detection of HIV-1 Transmission Clusters from Dried Blood Spots within a Universal Test-and-Treat Trial in East Africa
Source: Viruses. 2022 Jul 29;14(8):1673. doi: 10.3390/v14081673 (PMC9414799; doi:10.3390/v14081673)
Supplement: Supplementary file 1 [file viruses-14-01673-s001.zip › viruses-1744283-supplementary.pdf]

Detection of HIV-1 Transmission Clusters from Dried Blood Spots within a Universal Test-and-Treat Trial in East Africa

Supplementary Materials

Table S1. Estimated regression parameters, standard errors, z-values, and p-values for the logistic regression model used to determine whether certain characteristics made sequenced SEARCH participants more likely of being in a <1.5% genetic distance (GD) cluster.

	Estimate	Standard Error	z-value	p-value
Intercept	-19.681	1317.641	-0.015	0.988
“HIV-1 infection”Incident	0.581	0.399	1.459	0.145
“Region”Kenya	16.081	1317.641	0.012	0.990
“Region”Western Uganda	16.562	1317.641	0.013	0.990
“Occupation”High-risk informal sector	-17.104	1458.524	-0.012	0.991
“Occupation”Low-risk informal sector	-1.076	0.442	-2.436	0.015 *
“Occupation”No job or disabled	-0.454	1.105	-0.411	0.681
“Occupation”Other	-0.132	0.704	-0.188	0.851
“Group”Intervention	1.207	0.458	2.635	0.008 **

Null deviance: 237.52 with $df = 729$,
Residual deviance: 209.54 with $df = 721$,
AIC: 227.54

Table S2. Settings for tested BEAST runs. All runs were performed for 1 billion generations, with sampling every 10th generation. Entries in **bold** represent final, selected runs.

	n	*Substitution model* *	*Molecular clock* †	*Coalescent tree prior* ⁕
pol A1	265	SRD06	Uncorrelated relaxed log-normal	Constant
pol A1	265	SRD06	Uncorrelated relaxed log-normal	Exponential
pol A1	265	SRD06	Uncorrelated relaxed log-normal	SkyGrid 4:80
pol A1	265	SRD06	Uncorrelated relaxed log-normal	SkyGrid 5:70
pol A1	265	SRD06	Uncorrelated relaxed log-normal	SkyGrid 20:80
pol A1	273	SRD06	Uncorrelated relaxed log-normal	SkyGrid 20:80
***pol* A1**	**297**	**SRD06**	**Uncorrelated relaxed log-normal**	**SkyGrid 20:80**
pol D	98	GTR	Uncorrelated relaxed log-normal	SkyGrid 4:60
pol D	98	SRD06	Uncorrelated relaxed log-normal	SkyGrid 4:60
pol D	102	GTR	Uncorrelated relaxed log-normal	SkyGrid 4:60
pol D	102	SRD06	Uncorrelated relaxed log-normal	SkyGrid 4:60
pol D	102	GTR	Uncorrelated relaxed log-normal	Skyride
pol D	102	SRD06	Uncorrelated relaxed log-normal	Skyride
pol D	102	GTR	Uncorrelated relaxed log-normal + narrow, normally distributed truncated prior (0.003 ± 0.01, in [0.001, 0.01])	Skyride
***pol* D**	**102**	**SRD06**	**Uncorrelated relaxed log-normal + narrow, normally distributed truncated prior (0.003 ± 0.01, in [0.001, 0.01])**	**Skyride**
***gag* A1**	**339**	**SRD06**	**Uncorrelated relaxed log-normal**	**SkyGrid 20:80**
gag D	116	GTR	Uncorrelated relaxed log-normal + narrow, normally distributed truncated prior (0.003 ± 0.01, in [0.001, 0.01])	Skyride
***gag* D**	**116**	**SRD06**	**Uncorrelated relaxed log-normal + narrow, normally distributed truncated prior (0.003 ± 0.01, in [0.001, 0.01])**	**Skyride**

* The SRD06 substitution model consists of the HKY model [34] with four categories of rate heterogeneity ($\gamma + 4$) [35] and two codon position partitions that are parameterised separately: positions (1+2) and position 3. The GTR substitution model [51] also included the $\gamma + 4$ site rate heterogeneity model.

† The uncorrelated relaxed log-normal molecular clock, as described by [33].

⁕ Constant [52], exponential [53], Skyride [37] and SkyGrid [36] coalescent tree priors. For SkyGrid, the first number indicates the number of parameters, the second the time at last transition point (in years), see [31, 36].

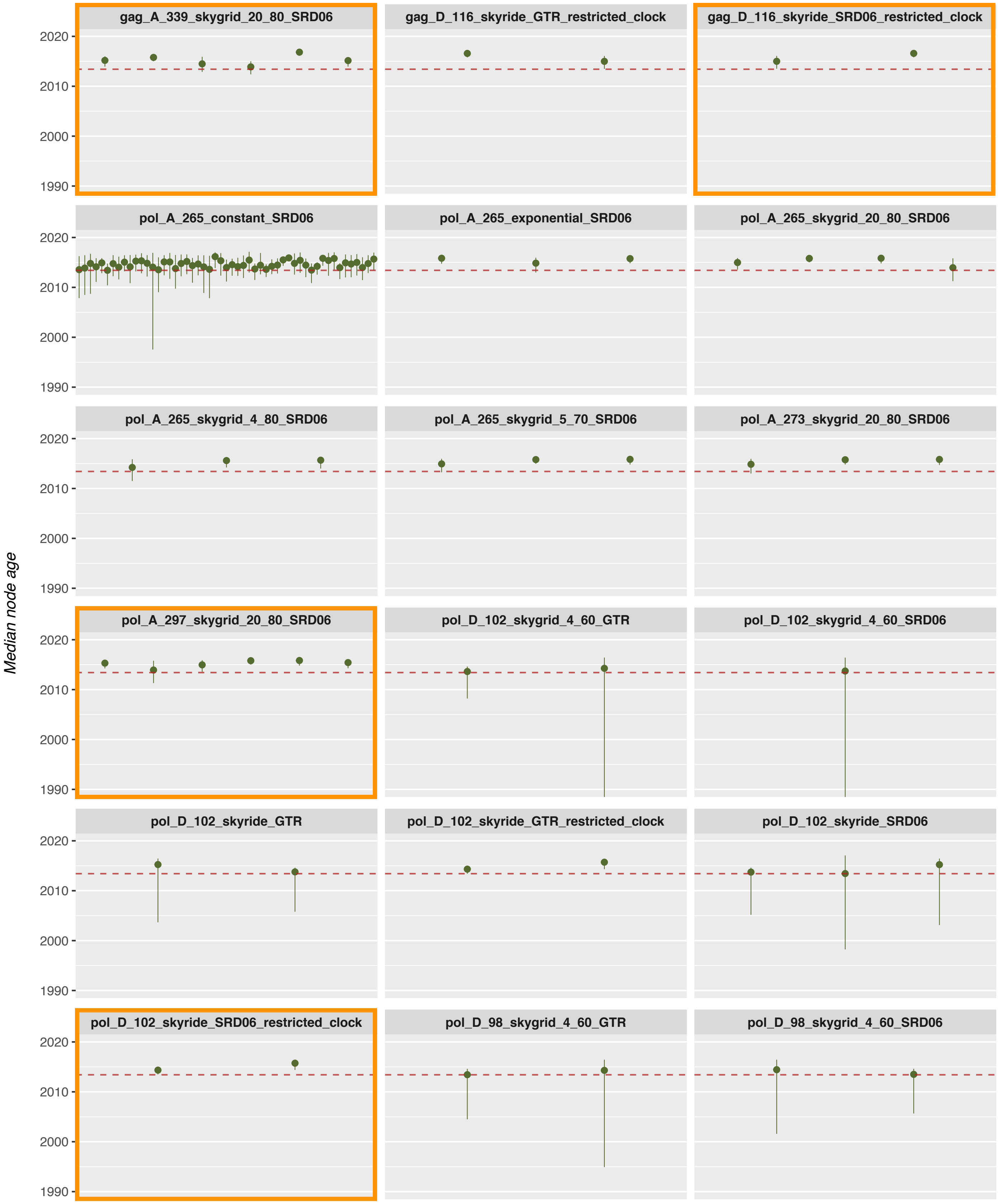

Figure S1. Median node ages and associated 95% highest posterior density (95% HPD) intervals for SEARCH-incident nodes (nodes with an age after the start of the SEARCH trial, June 2013, date cut-off indicated by the dashed red line). Highlighted plots indicate the final four runs selected for analysis (those that minimised the width of the 95% HPD intervals, minimising the error surrounding internal node age estimates). Note that for some nodes, the 95% HPD intervals extend outside of the plot limits. Each plot is labelled using the following notation: [gene]_[subtype]_[sequences]_[tree prior]_[substitution model]_[clock changes, if applicable]

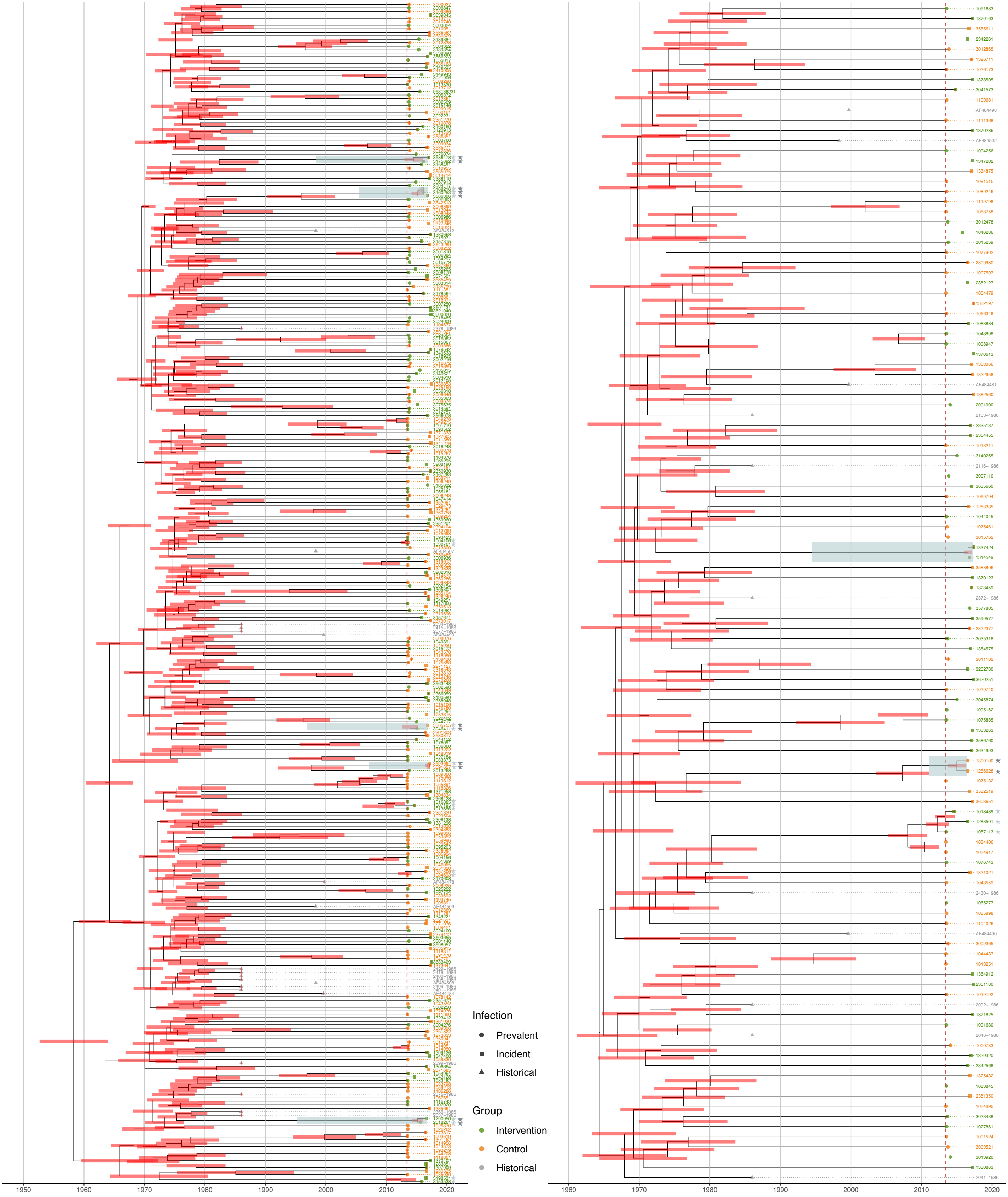

gag **subtype A1 (*n* = 339)**

gag **subtype D (*n* = 116)**

Figure S2. Maximum clade credibility trees with median node ages for *gag* A1 (left) and *gag* D (right) SEARCH trial sequences. Circular, square and triangular tips represent prevalent, incident and historical sequences, respectively; green, orange and grey tips represent intervention, control and historical sequences, respectively; red bars denote the 95% highest posterior density interval for a node; blue-grey shaded boxes highlight clusters with a median age after the start of the SEARCH trial (June 2013); light stars denote sequences in the final <1.5% GD clusters; dark stars denote sequences in the final phylodynamic SEARCH-incident clusters.

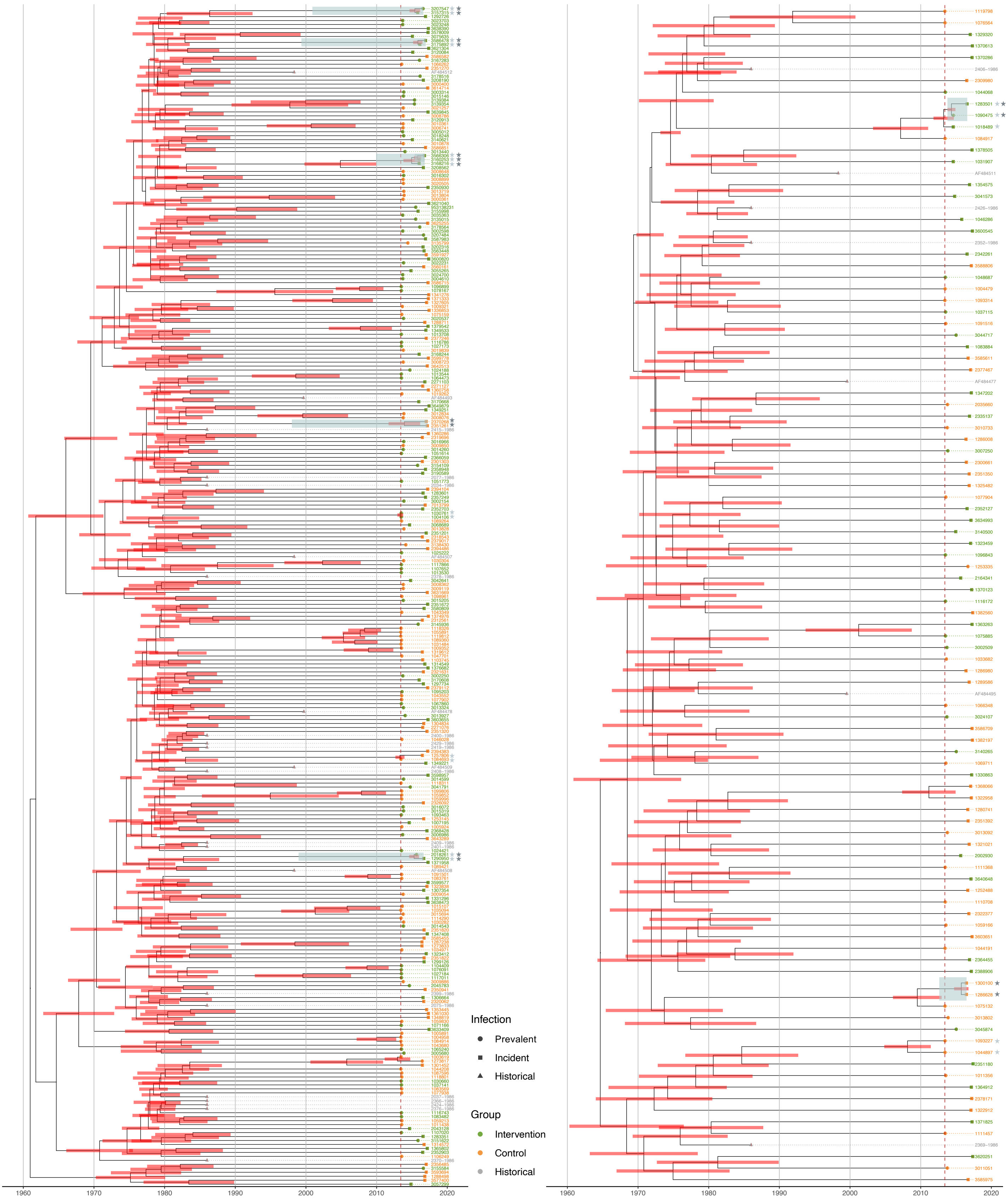

***pol* subtype A1 (*n* = 297)** ***pol* subtype D (*n* = 102)**

Figure S3. Maximum clade credibility trees with median node ages for *pol* A1 (left) and *pol* D (right) SEARCH trial sequences. Circular, square and triangular tips represent prevalent, incident and historical sequences, respectively; green, orange and grey tips represent intervention, control and historical sequences, respectively; red bars denote the 95% highest posterior density interval for a node; blue-grey shaded boxes highlight clusters with a median age after the start of the SEARCH trial (June 2013); light stars denote sequences in the final <1.5% GD clusters; dark stars denote sequences in the final phylodynamic SEARCH-incident clusters.

Table S3. Estimated regression parameters, standard errors, z-values, and p-values for the logistic regression model used to determine whether certain characteristics made sequenced SEARCH participants more likely of being in SEARCH-incident clusters.

	Estimate	Standard Error	z-value	p-value
Intercept	-3.814	0.633	-6.024	1.7×10^{-9} ***
"HIV-1 infection"Incident	2.051	0.646	3.174	0.0015 **
"Occupation"High-risk informal sector	-16.999	1447.060	-0.012	0.991
"Occupation"Low-risk informal sector	-1.836	0.545	-3.367	0.0008 ***
"Occupation"No job or disabled	-17.256	2762.249	-0.006	0.995
"Occupation"Other	-0.224	0.828	-0.271	0.787

Null deviance: 161.44 with $df = 729$,
Residual deviance: 130.79 with $df = 724$,
AIC: 142.79

Table S4. Characteristics of sequenced SEARCH trial participants, by trial arm (*n* = 745).

	Intervention ***n (%)***	***Control*** ***n (%)***	***NA*** ***n (%)***	***Total*** ***n (%)***
All	348 (46.7)	395 (53)	2 (0.3)	745 (100)
Gender				
Female	199 (57.2)	209 (52.9)	2 (100)	410 (55)
Male	149 (42.8)	186 (47.1)	0	335 (45)
Age category				
(χ^2 test *p*-value = 0.01)				
15 – 20 years	27 (7.8)	12 (3)	0	39 (5.2)
21– 49 years	266 (76.4)	328 (83)	0	594 (79.7)
≥ 50 years	47 (13.5)	50 (12.7)	0	97 (13)
NA	8 (2.3)	5 (1.3)	2 (100)	15 (2)
Occupation *				
(χ^2 test *p*-value = 0.009)				
Formal sector	62 (17.8)	60 (15.2)	0	122 (16.4)
High-risk informal sector	25 (7.2)	25 (6.3)	0	50 (6.7)
Low-risk informal sector	221 (63.5)	291 (73.7)	0	512 (68.7)
Other	23 (6.6)	9 (2.3)	0	32 (4.3)
No job or disabled	9 (2.6)	5 (1.3)	0	14 (1.9)
NA	8 (2.3)	5 (1.3)	2 (100)	15 (2)
HIV-1 infection category †				
(χ^2 test *p*-value = 0.03)				
Prevalent	204 (58.6)	263 (66.6)	2 (100)	469 (63)
Incident	144 (41.4)	132 (33.3)	0	276 (37)
Region				
(χ^2 test *p*-value = 6×10^{-9})				
Western Uganda	142 (40.8)	235 (59.5)	0	377 (50.6)
Eastern Uganda	22 (6.3)	39 (9.9)	0	61 (8.2)
Kenya	184 (52.9)	121 (30.6)	0	305 (40.9)
NA	0	0	2 (100)	2 (0.3)

* A *formal sector* occupation was defined as a teacher, student, government worker, military worker, health worker, or factory worker. A *high-risk informal sector* occupation was defined as a fishmonger, fisher, bar owner, bar worker, transportation worker, or factory worker. A *low-risk informal sector* occupation was defined as a farmer, shopkeeper, market vendor, hotel worker, homemaker, household worker, construction worker, or miner.

† *HIV-1 infection category* defined as *prevalent* if HIV-1 positive at SEARCH trial baseline and *incident* if HIV-1 negative at baseline but seroconversion detected during the trial.

Table S5. Characteristics of sequenced SEARCH trial participants, according to HIV-1 infection category[†] (n = 745).

	Prevalent n (%)	***Incident n (%)***	***Total n (%)***
All	469 (62.9)	276 (37.1)	745 (100)
Gender			
Female	260 (55.4)	150 (54.3)	410 (55)
Male	209 (44.6)	126 (45.7)	335 (45)
Age category			
(χ^2 test p-value = 1.5×10^{-4})			
15 – 20 years	15 (3.2)	24 (8.7)	39 (5.2)
21– 49 years	365 (77.8)	229 (83)	594 (79.7)
≥ 50 years	74 (15.8)	23 (8.3)	97 (13)
NA	15 (3.2)	0	15 (2)
Occupation *			
Formal sector	71 (15.1)	51 (18.5)	122 (16.4)
High-risk informal sector	29 (6.2)	21 (7.6)	50 (6.7)
Low-risk informal sector	330 (70.4)	182 (65.9)	512 (68.7)
Other	18 (3.8)	14 (5.1)	32 (4.3)
No job or disabled	6 (1.3)	8 (2.9)	14 (1.9)
NA	15 (3.2)	0	15 (2)
Trial arm			
(χ^2 test p-value = 0.03)			
Intervention	204 (43.5)	144 (52.2)	348 (46.7)
Control	263 (56.1)	132 (47.8)	295 (53)
NA	2 (0.4)	0	2 (0.3)
Region			
(χ^2 test p-value < 2×10^{-16})			
Western Uganda	263 (56.1)	114 (41.3)	377 (50.6)
Eastern Uganda	5 (1.1)	56 (20.3)	61 (8.2)
Kenya	199 (42.4)	106 (38.4)	305 (40.9)
NA	2 (0.4)	0	2 (0.3)

* A *formal sector* occupation was defined as a teacher, student, government worker, military worker, health worker, or factory worker. A *high-risk informal sector* occupation was defined as a fishmonger, fisher, bar owner, bar worker, transportation worker, or factory worker. A *low-risk informal sector* occupation was defined as a farmer, shopkeeper, market vendor, hotel worker, homemaker, household worker, construction worker, or miner.

[†] *HIV-1 infection category* defined as *prevalent* if HIV-1 positive at SEARCH trial baseline and *incident* if HIV-1 negative at baseline but seroconversion detected during the trial.

Table S6. SEARCH trial sequences with low- to high-level NRTI/NNRTI resistance, according to WHO standards for resistance classification, alongside NRTI/NNRTI-associated mutations.

Sequence ID	NRTI Resistance	NNRTI Resistance	NRTI-associated Mutations	NNRTI-associated Mutations
1005830		High		Y188C
1013708		High		K103N
1018489		High		K103N
1030282	High	High	M184V	K103N
1032229	High	High	M184V	K103N,H221Y
1037115		Intermediate		K101E,E138A
1038481	High	High	K65R,M184V	K101E,E138A,G190C
1043997	High	High	M184V,T215F	K103N,Y181C,G190A
1047414		High		K103KN,V108VI,Y181YC
1055948	High	High	M184V	K103N
1059213		High		A98AG,K103KN,F227FL
1067860	Low		T215TI	
1087596		High		K103N
1087677	High	High	A62V,K65R,M184V	V106M,V179E
1090475		High		K103N
1091340	Low		M41L	E138K
1093314		High		K103N
1099160	High	High	M41L,E44D,D67N,M184V,L210W,T215Y	V106A,P225H
1103745		High		K103KN
1111457		High		Y181C
1252723	Intermediate	High	D67N,L210GW,T215S	K103N,Y181C,G190A,H221Y
1273817		High		K103N
1280741		High		K103N
1283501		High		K103N
1289586		High		Y188L
1297734		Intermediate		A98G
1331296	High	High	A62V,K70E,V75I,M184V	K103S,G190A
1333591		High		K103N
1347408		High		K103N
1370402		Low		E138G,V179E
1370750		High		K103KN,V106VIM
1371333		High		K103N
2001000	High	Intermediate	M184I	M230I
2271127	Low	High	T215D	V108I,Y181C,H221Y
2309980	Low	High	T215D	K103N,H221Y,M230L,L234I
2318543		High		K103N,V179T,Y188F
2321631		High		V106VA,E138AGV,G190A
2356485	High	High	K65KR,D67N,K70KR,M184V,K219QR	K103N,P225H
2364455		High		Y181C,H221Y
2410005		High		K103N
3000361		High		K103N
3000751		High		K103N,Y181YC
3008723	High	High	M184V	E138A,Y181I
3008786		High		Y188L
3009054	High	High	K65R,D67N,V75I,F77L,F116Y,Q151M,M184V,K219E	K103N,Y181C,G190A
3009119		High		A98G,Y181C
3010275	High	High	D67G,K70T,T215F	A98G,V108I,Y181F
3010878		High		K103N
3011104	High	High	M41L,V75I,M184V,T215Y	A98G,K103N,E138Q
3013810		High		K103N
3013832		High		K103N
3015319	High	Intermediate	M184I	M230I
3016966	High	High	M184V	K103N
3022231	High	High	M41L,M184V,L210W,T215F	A98G,K103S
3055265		High		K103N
3075635		High		K103N
3139354		High		K103N
3139384	High	High	M184MIV,T215CDGY	Y181C
3207547		High		K103N
3208066	Low		M41ML	
3560161		High		K103N
3563448		High		G190A
3587360		High		K103N
3598957		High		K103N
3600820		High		K103N
3634441	High	High	K70N,M184V	V106I,Y188L,P225H
3640648		Low		V108I
3649879	High	High	M184V	G190A
3591927		High		K103N,H221Y
